# Longitudinal CT, MRI, and 18F-FDG PET/CT Imaging Findings of Peliosis Hepatis: A Case Report

**DOI:** 10.7759/cureus.62997

**Published:** 2024-06-23

**Authors:** Yuki Yamada, Ryo Kurokawa, Mariko Kurokawa, Rin Tsujimoto, Arika Shimura, Hiroaki Maki, Atsushi Kondo, Osamu Abe

**Affiliations:** 1 Radiology, The University of Tokyo, Tokyo, JPN; 2 Hematology and Oncology, The University of Tokyo, Tokyo, JPN; 3 Pathology, The University of Tokyo, Tokyo, JPN

**Keywords:** positron emission tomography, magnetic resonance imaging, computed tomography, radiology, peliosis hepatis

## Abstract

Peliosis hepatis (PH) is a rare benign vascular condition characterized by sinusoidal dilatation and the presence of blood-filled spaces within the liver. PH is often clinically asymptomatic and is discovered incidentally. It presents a clinical challenge as its imaging findings frequently mimic other pathologies, including primary or secondary malignancies and abscesses. In this article, we present a case of a 73-year-old woman with a history of recurrent tongue cancer treated by surgery and chemoradiotherapy, and concurrent multiple myeloma, in whom PH was incidentally discovered. Based on computed tomography, magnetic resonance imaging, and 18F-fluorodeoxyglucose positron emission tomography (18F-FDG PET) imaging findings prior to biopsy, PH was diagnosed, and pathologically confirmed. Follow-up computed tomography five months after the discontinuation of raloxifene hydrochloride, a selective estrogen receptor modulator and a suspected drug causing PH, the regression of PH lesions was observed.

## Introduction

Peliosis hepatis (PH) is a rare benign vascular condition characterized by sinusoidal dilatation and the presence of blood-filled cystic cavities within the liver parenchyma. The term is derived from the Greek word “Pelios”, which means “reddish” or “bluish.” The etiology of PH has not been fully understood, but PH is associated with medications, infections, malignancies, and genetic disorders [1]. PH often remains clinically asymptomatic, leading to incidental detection. The condition is not well-recognized among clinicians, and its imaging findings can be diverse, making diagnosis challenging. Consequently, it is frequently misdiagnosed as liver metastases, primary liver tumors, or abscesses [2,3]. Correct diagnosis of PH by imaging is important for appropriate management [1]. Furthermore, few cases have been able to trace changes in imaging findings to the course of PH enlargement over time and reduction with discontinuation of the suspected causative drug [4]. In this article, we present a case of a 73-year-old woman with a history of tongue cancer surgery, chemoradiotherapy, and concurrent multiple myeloma, in whom PH was incidentally discovered. PH was successfully diagnosed based on computed tomography (CT), magnetic resonance imaging (MRI), and 18F-fluorodeoxyglucose positron emission tomography (FDG-PET)/CT imaging findings prior to biopsy, and CT imaging findings of the course of PH enlargement over time and reduction with discontinuation of the suspected drug was confirmed.

## Case presentation

**Figure 1:**
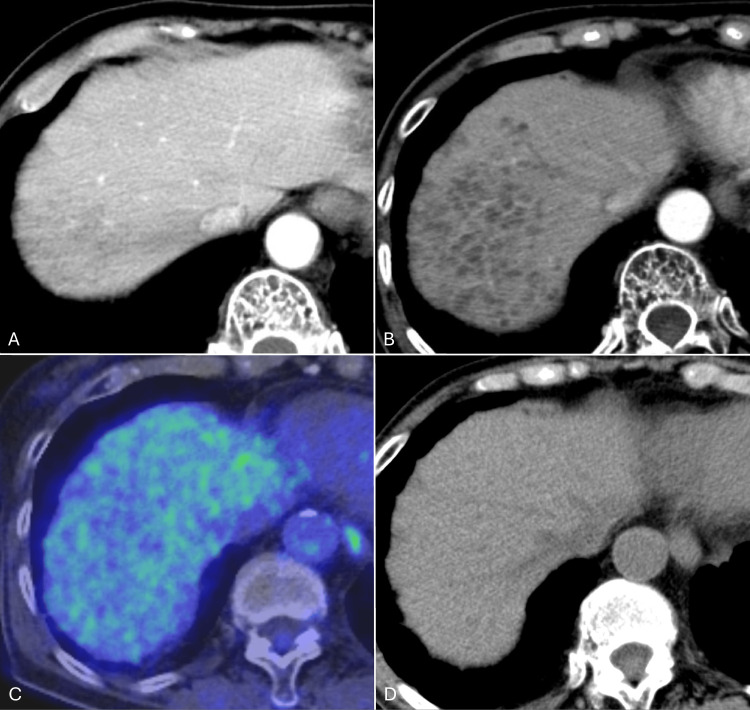
18F-FDG PET/CT and longitudinal CT images of peliosis hepatis A: Contrast-enhanced CT performed one year and four months ago showed multiple cystic lesions in the right hepatic lobe. B: Contrast-enhanced CT shows increased cystic lesions. C: 18F-FDG PET/CT shows decreased FDG-uptake areas in the lesions. D: Five months after the discontinuation of the suspected causative drug shows regression of the cystic lesions on nonenhanced CT. 18F-FDG PET/CT, 18F-fluorodeoxyglucose positron emission tomography/computed tomography

A 73-year-old woman, who had undergone surgery for tongue cancer seven years prior and a second surgery for recurrent tongue cancer four years ago followed by postoperative chemoradiotherapy, presented to our hospital for evaluation of incidentally discovered multiple cystic lesions in the liver. Two months earlier, she experienced back pain, and a plain X-ray at a local clinic revealed multiple vertebral fractures. Peripheral blood tests suggested multiple myeloma with immunoglobulin G-lambda type positivity, and she was scheduled for further evaluation at the Department of Hematology and Oncology of our hospital. A follow-up contrast-enhanced CT for treated tongue cancer revealed multiple hypovascular nodules in the right hepatic lobe (Figure 1).

**Figure 2:**
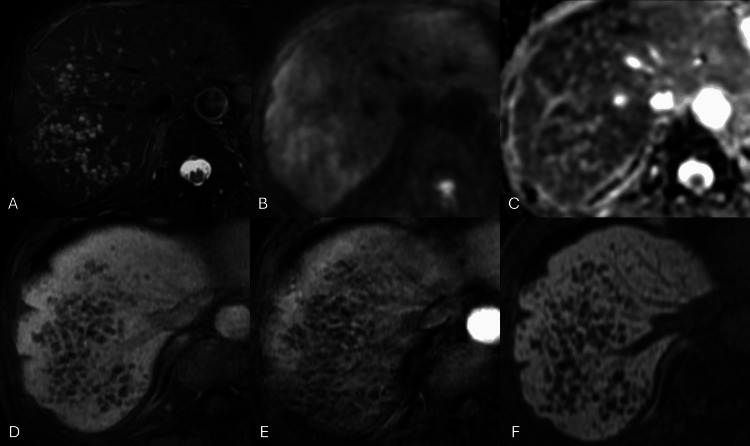
MRI of peliosis hepatis There are multiple cystic lesions with high intensity on fat-suppressed T2-weighted imaging with fluid-fluid levels (A) and absence of restricted diffusion (B: diffusion-weighted imaging, C: apparent diffusion coefficient map). Fat-suppressed T1-weighted imaging shows hypointensity (D) without contrast enhancement in the arterial phase (E) or EOB uptake in the hepatobiliary phase (F) on dynamic MRI using Gd-EOB-DTPA. MRI, magnetic resonance imaging; Gd-EOB-DTPA, gadolinium ethoxybenzyl diethylenetriaminepentaacetic acid

18F-FDG PET/CT for evaluation of cystic liver diseases and for screening potential metastasis of the treated tongue cancer revealed no increased FDG uptake on the lesions. Gradual increase and expansion over time were confirmed compared to the CT performed one year and four months earlier. Dynamic contrast-enhanced MRI of the upper abdomen using gadolinium ethoxybenzyl diethylenetriaminepentaacetic acid (Gd-EOB-DTPA) showed multiple subcentimeter cystic lesions with fluid-fluid levels in the right hepatic lobe without restricted diffusion or contrast enhancement (Figure 2).

The patient's medications included telmisartan and amlodipine for hypertension, eldecalcitol capsules, raloxifene hydrochloride tablets, teriparatide tablets for osteoporosis, and limaprost alfadex for chronic pain. Blood tests showed elevated immunoglobulin G with a low kappa/lambda ratio, normal tumor markers, and no evidence of inflammation (Table 1).

**Table 1:** Blood test results Serum immunoglobulin G was abnormally elevated, with a low free light chain kappa/ lambda ratio, suggesting immunoglobulin G lambda-type multiple myeloma. There is no elevation in inflammatory markers, and no increase in tumor markers is observed.

Parameters	Value	Normal range
Total protein	7.9 g/dL	6.6–8.1 g/dL
Albumin	3.4 g/dL	4.1–5.1 g/dL
Immunoglobulin A	58 mg/dL	93–393 mg/dL
Immunoglobulin G	3,041 mg/dL	861–1,747 mg/dL
Immunoglobulin M	20 mg/dL	50–269 mg/dL
Kappa/Lambda ratio	0.03 (9.6 / 368 mg/dL)	0.26–1.65
β2-microglobulin	3.0 mg/L	0.9–2.0 mg/L
β-D glucan	9.8 pg/mL	0–20 pg/mL
C-reactive protein	0.03 mg/dL	0–0.3 mg/dL
White blood cell	4,900 /µL	3,300–8,600 /µL
Hemoglobin	9.3 g/dL	11.6–14.8 g/dL
Platelets	269,000 /µL	158,000–348,000 /µL
Alpha fetoprotein	1.6 ng/mL	0–9.9 ng/mL
PIVKA-II	17 mAu/mL	0–39 mAu/mL
Carcinoembryonic antigen	1.2 ng/mL	0–4.9 ng/mL
Carbohydrate antigen 19-9	9 U/mL	0–36 U/mL

**Figure 3:**
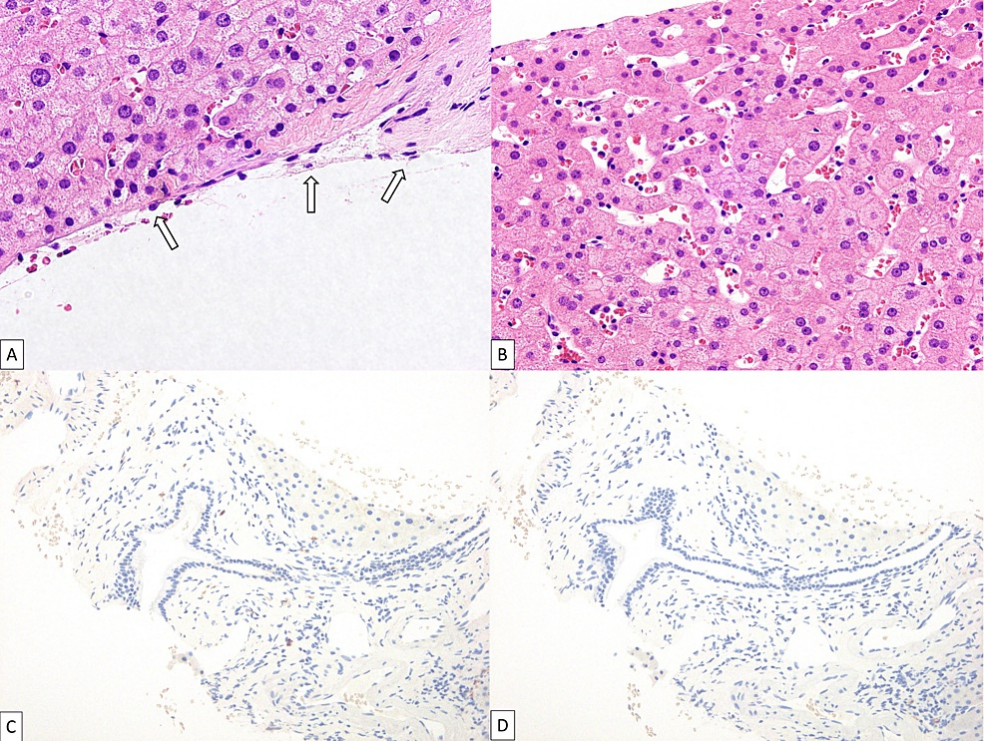
Histopathology of phlebectatic peliosis hepatis Hematoxylin and eosin stain reveals the formation of cavities with smooth surfaces lined by endothelial cells (arrows in A, x600). Sinusoidal dilation and blood pooling are noted (B, x400). No light chain restriction is observed by kappa (C) and lambda (D) in situ hybridization.

Radiologically, the lesions showed multiple clustered cysts with low attenuation on CT, hyperdensity with fluid-fluid levels on T2-weighted imaging, hypodensity on T1-weighted imaging, increased diffusion, no contrast enhancement on MRI, and the absence of FDG uptake. Combined with the clinical information, the following diseases were raised as differential diagnoses: metastases with or without primary liver cancer, hepatic infiltration of multiple myeloma, amyloidosis associated with multiple myeloma, and abscesses. The strong cystic degeneration was considered atypical for metastases from the treated tongue cancer or other primary. Moreover, the absence of diffusion restriction and FDG uptake on imaging further lowered the possibility. There was no evidence suggesting the existence of a potential primary tumor other than the liver on FDG-PET/CT. The morphology of multiple clustered cystic lesions argued against primary liver tumors, hepatic infiltration of multiple myeloma, and amyloidosis. The presence of the lesion for one year and four months prior to presentation ruled out the possibility of abscesses. Ultimately, the most likely possibility of PH was reported in the diagnostic imaging report. A liver biopsy was performed, and the hepatic lesions were histopathologically consistent with phlebectatic PH (Figure 3), ruling out primary liver tumors, metastatic malignancies, infections, and amyloidosis.

A bone marrow biopsy confirmed the diagnosis of multiple myeloma, and DaraRd therapy was initiated. Due to the duration of administration and type of drug, raloxifene hydrochloride (selective estrogen receptor modulator) was suspected as the causative drug for PH and was discontinued four months later. A CT scan performed five months after discontinuation (nine months after the initial CT, MRI, and PET/CT) confirmed the regression of PH (Figure 1).

## Discussion

We present a case of a 73-year-old woman with a history of recurrent tongue cancer treated by surgery and chemoradiotherapy and concurrent multiple myeloma, in whom multiple cystic lesions of the liver were incidentally discovered. Differential diagnoses included metastases and liver invasion of the multiple myeloma, but the radiological perioperative imaging diagnosis of peliosis hepatis (PH) using CT, MRI, and 18F-FDG PET/CT was correct, and the biopsy confirmed it. Additionally, the disappearance of the lesions was confirmed after the suspected drug was discontinued.

PH is a rare vascular condition characterized by focal or diffuse blood-filled cavities within the liver parenchyma. The cysts are lined by endothelial cells in the phlebectatic subtype while they lack an endothelial lining in the parenchymal subtype, and the cystic spaces can vary in size from 1 mm to several centimeters [3,5]. Definitive diagnosis requires a liver biopsy showing the characteristic histological features. Clinically, patients with PH are usually asymptomatic, and thus PH is usually discovered incidentally on imaging or autopsy [5]. Some patients may present with hepatomegaly, abdominal pain, jaundice, portal hypertension, liver dysfunction, and hemoperitoneum due to rupture of the cysts [4-6]. PH is frequently associated with conditions including various kinds of drugs (e.g., anabolic steroids, corticosteroids, immunosuppressants, selective estrogen receptor modulators, and androgen), hematological malignancies, and chronic infections (e.g., tuberculosis, human immunodeficiency virus) [2]. According to a systematic review study of 49 patients with PH, the most frequently associated condition was drug-induced PH (35%) [3]. In the present case, raloxifene hydrochloride (a selective estrogen receptor modulator) was considered a causative drug for PH, because selective estrogen receptor modulators have been known to trigger PH through sinusoidal epithelial damage, glutathione depletion, idiosyncratic reactions to metabolites, estrogenic effects, or post-sinusoidal obstruction [1]. The regression of multiple cystic spaces after the discontinuation of the drug supported this theory.

On imaging examinations, PH lesions usually appear as single or multiple areas of varying-sized cysts with hypoattenuation to liver parenchyma on unenhanced CT, hyperintense to liver parenchyma or heterogeneous signal intensity on T2-weighted imaging, and hypointense to liver parenchyma or heterogeneous signal intensity on T1-weighted imaging (Table 2) [4].

**Table 2:** CT, MRI, and 18F-FDG PET imaging findings of peliosis hepatis * The percentage was cited from Calistri et al. [3]. CT, computed tomography; MRI, magnetic resonance imaging; 18F-FDG PET, 18F-fluorodeoxyglucose positron emission tomography

Modalities and sequences	Imaging findings
Unenhanced CT	Hypoattenuation
Dynamic contrast-enhanced CT	Vessel-like enhancement and the target sign on arterial phase
	Centrifugal or centripetal enhancement on portal venous phase
	Diffuse homogeneous enhancement in the phlebectatic type of peliosis hepatis on delayed phase
T2-weighted MR imaging	High or heterogeneous intensity
T1-weighted MR imaging	Low or heterogeneous intensity
Diffusion-weighted MR imaging	High or isointensity
Apparent diffusion coefficient	High
Dynamic contrast-enhanced MRI*	Heterogeneous (45.1%)
	Rim-like centripetal (23.5%)
	Centrifugal (13.7%)
	Homogeneously high intensity (10.0%)
	Persistently low intensity (7.8%)
18F-FDG PET	Hyper or hypometabolism

Other CT attenuation and MRI signal intensities can be observed depending on the status of the blood component. Diffusion-weighted imaging shows hyperintensity or isointensity with a high apparent diffusion coefficient. On dynamic contrast-enhanced MRI, PH lesions show heterogeneous (45.1%), rim-like centripetal (23.5%), centrifugal (13.7%), homogeneously high (10.0%), or persistently low intensity (7.8%) [3]. Thrombus within the cavities, hemorrhage, and calcifications may be found. Limited case reports suggested hypometabolism or hypermetabolism on 18F-FDG PET [7,8]. In the present case, PH lesions showed hypointense on enhanced CT, mainly hyperintensity with fluid-fluid levels on T2-weighted imaging, hypointensity on T1-weighted imaging, persistent hypointensity without contrast enhancement, increased diffusion, hypometabolism on 18F-FDG PET. The absence of contrast enhancement was a rare pattern, but other imaging findings in the present case were consistent with the literature. Due to the complicated history of the patient, multiple clinical departments were initially involved in the present case, but the imaging diagnosis of PH, which was promptly confirmed pathologically with a liver biopsy, allowed appropriate management of PH with discontinuation of the suspected drug. For a patient who can be monitored for several months, follow-up with CT alone without the need for liver biopsy could have been a viable option. Differentiation from other malignancies and abscesses avoided unnecessary and invasive measures, such as liver resection and anticancer treatment, leading to appropriate treatment for the patient and preventing waste of healthcare resources.

## Conclusions

We presented a case of PH incidentally discovered and successfully diagnosed in a patient with a history of treated recurrent tongue cancer and concomitant multiple myeloma. Furthermore, we were able to follow the patient until the regression of the lesions after discontinuing the suspected causative drug. Differentiating PH from its mimickers, such as primary and secondary liver tumors or abscesses, often poses clinical and radiological challenges. However, a detailed interpretation of CT, MRI, and 18F-FDG PET/CT findings, combined with the temporal changes of the lesions, may lead to an accurate diagnosis of PH. Although there have been previous reports on the typical imaging findings of PH, studies that include follow-up of the temporal changes in imaging findings, including after the discontinuation of the suspected causative drug, are scarce. Understanding the imaging findings and temporal changes reported in this article is useful for the appropriate radiological diagnosis and clinical management of patients with PH.
